# Impact of fine motor skills acquisition and psychological factors on sex-specific performance in early interventional radiology training

**DOI:** 10.3389/fmed.2025.1638221

**Published:** 2025-12-05

**Authors:** Sebastian R. Reder, Katja Petrowski, Katja U. Beiser, Mario A. Abello, Naureen Keric, Katya Hoffmannbeck Heitkötter, Ahmed E. Othman, Laura Leukert, Nils F. Grauhan, Marc A. Brockmann, Carolin Brockmann

**Affiliations:** 1 Department of Neuroradiology, University Medical Center, Johannes Gutenberg-University of Mainz, Mainz, Germany; 2 Department of Medical Psychology and Medical Sociology, University Medical Center of the Johannes Gutenberg-University Mainz, Mainz, Germany; 3 Department of Neurosurgery, University Hospital of Schleswig-Holstein, Campus Lübeck, Lübeck, Germany

**Keywords:** interventional radiology, sex differences, medical training, motor skills, psychological factor, healthcare education and training

## Abstract

**Objectives:**

To explore the impact of self-reported motor skills on sex-specific training outcome in early interventional radiology (INR).

**Materials and methods:**

Based on the population of Reder et al., the study assessed the frequency, duration, and intensity of manual-focused activities, encompassing both non-professional and professional engagements (64 participants; 25 women), using the NASA Task Load Index (NASA-TLX) scores for mental workload following a standardized INR training session for basic techniques. Spearman’s correlation and multiple regression analyses with backward elimination and stepwise variable selection were conducted.

**Results:**

Sex-specific significant differences exist for predicting objective performance (OP) based on lifetime-developed hand focus. Leisure activities revealed a positive correlation with OP in men (*β* = 0.526; *p* = 0.001), unlike women. Conversely, profession-based activities correlated negatively with OP in men (*β* = −0.579; *p* = 0.022). In women, non-profession-based and profession-based activities did not significantly correlate with achieved OP. However, self-assessed performance (NASA-TLX) correlated with OP in women (*β* > 0.5 and *p* < 0.01 each). In men, cumulative hand focus (or fine motor skills) is crucial for success, influenced negatively by excessive profession-related work load (*p* = 0.025). In women, only self-assessment was responsible for objectively determined success or failure.

**Conclusion:**

In INR training, women might benefit more from external motivation, mental training, and empowerment, while men might profit more from physical training resembling observational learning. Considering the observed negative impact of excessive profession-related work load on men, a balanced lifestyle might lead to better outcomes related to fine motor skill demanding tasks.

## Highlights

- Identifying sex-specific learning strategies to improve interventional radiology training.
- Women’s performance is influenced by self-assessment and psychological factors.
- Men benefit from varied, experience-based learning, including leisure activities.
- High professional-based workload reduced objective performance.
- Women might benefit more from external motivation; men from observational learning.

## Introduction

Accurate self-assessment of performance is an essential component of professional development, yet significant gender differences in this regard have been observed (1–6). Research indicates that women tend to have a more accurate understanding of their own abilities, whereas men often overestimate their performance (5). This discrepancy, especially in high-stakes fields such as medicine, could have critical implications for both training and outcomes (2, 5, 7–11). Despite these findings, women remain underrepresented in surgical and interventional specialties (5, 12, 13), including interventional radiology (IR), raising the question of whether women’s more accurate self-assessment could contribute to their lower participation (1, 5, 6, 14, 15). This may be linked to factors such as perceived barriers or a lack of confidence, despite evidence of high competence. This disparity is attributed to several factors, including potential misconceptions about the field among medical students and female physicians, likely due to issues related to pregnancy, sex-specific dynamics in teamwork, and insufficient mentoring (15). Particularly, the concept of empowerment is multifaceted and influenced by numerous factors (16).

The manuscript “Gender differences in self-assessed performance and stress level during training of basic interventional radiology maneuvers” by Reder et al. (5), primarily focused on the female perspective, explored the impact of self-assessment and stress on performance during simulated interventional radiology tasks. However, the male perspective was not fully addressed, leaving a significant gap in understanding. Unresolved questions emerged regarding why men seemed to struggle with accurately assessing their performance, why higher reported stress levels correlated with poorer performance in men, and why stress appeared to have no such correlation in women. These findings prompted further investigation into these gender-specific disparities.

For this purpose, the previously published dataset and methodology of Reder et al. (5) was revisited using refined methodology (5), wherein individual cumulative fine motor skills acquired over the course of life were attempted to be objectively quantified and correlated with self-perception regarding objectively performed tasks. A positive effect of cumulative fine motor skills (or hand focus) over the individuals’ lifespan on the objective performance parameters of the experiments was hypothesized.

## Materials and methods

The baseline population and methodology for data acquisition (including training scenarios, experimental setting, objective and subjective data acquisition) have been described and published already in a previous study by Reder et al. (5). Data known from this study are unequivocally indicated and reported as Supplementary material.

### Ethics and informed consent

The study was conducted in accordance with the ethical standards as laid down in the 1964 Declaration of Helsinki and its later amendments or comparable ethical standards. As participation was voluntarily, posed no biological risks, participants were informed about data protection policy and gave written informed consent, and data were published anonymously, the Ethics Committee of the Landesärztekammer Rheinland-Pfalz waived issuing a statement.

### Population recruitment and participation criteria

Sixty-four participants (26 females and 38 males; see *“Sample Size Calcualtion”*) with no prior experience in INR techniques were recruited in 2020 from a University Medical Center by the same male MD student (three personally known by the recruiter) (5). One female participant, unknown to the recruiter, withdrew during the second task due to perceived task difficulty (5).

### Standardized training

Standardized training preceded the independent execution of simulated endovascular procedures by all 64 participants, as stated out by Reder et al. (5). The study encompassed four tasks of varying difficulty levels, with the third task (Task 3) being the most challenging, while the first and fourth tasks were the least demanding (5).

### Catheter model and interventional tasks

A silicone replica (NST00V02 #5117; United Biologics Inc.) representing the life-sized vascular anatomy from the femoral artery to the superior sagittal sinus and filled with a destilled water-baby shampoo mixture (100:1 ratio) was utilized for the experiments (Supplementary Figure 1A) (5). The experiments started from a standardized catheter position in the upper descending aorta (Asterisk* in Supplementary Figures 1B,D) (5).

The aims were as follows (Supplementary Figures 1B–E), as described by Reder et al. (5):

- Task 1: to explore the V2-segment of the left vertebral (VA).
- Task 2: to navigate a microcatheter into a tip-aneurysm of the basilar artery (BA).
- Task 3: to probe the right internal carotid artery (ICA).
- Task 4: to navigate a microwire tip into an ICA-sidewall aneurysm.

After successful completion of the experimental setting, the participants were directed to fill out a questionnaire, incorporating the NASA-Task Load Index (5).

### Objective study parameters

The subsequent parameters were acquired prospectively and obtained following for each task (5):

- Time [s]: Duration to finish each task
- Pathway [cm]: distance traversed by the catheter within the vascular model using the freely accessible software “Viana.net“(Free Software Foundation Inc.)
- Number of movements (“NoM”) within predefined regions of interest (“ROI”)
- Number of attempts (“NoA”) defined as a forward motion followed by a corrective backward motion within an ROI, categorized as one “attempt”
- Throughout the experiment, all participants had the option to seek standardized assistance if needed, with awareness that any aid sought would be documented (5). The frequency of requests for assistance and the duration until participants comprehended assistance were recorded (5). The time was paused during this period (5).

The Total Objective Performance (or “TOP”) was determined using the z-transformed variables of total time, distance, NoM, and NoA. The z-transformation reduced values to their distance from the mean in terms of the standard deviations of the given dataset. Consequently, SI units (such as centimeters or seconds) became insignificant, ensuring value comparability, and allowing a sum score from the four mentioned z-transformed objective values. Since z-scores would indicate below-average (negative values) and above-average (positive values) performance depending on whether the values are low or high, the objective overall performance was multiplied by −1. This allowed “good values” (e.g., low time or short pathways) to be defined as “good,” whereas without this correction, low values would be defined by the z-score as below average, and thus, interpreted as “bad.” The sum of the adjusted values was defined as the TOP.

### Subjective study parameters

Subjective parameters encompassed self-evaluated performance, stress level, and physical efforts (5). In contrast to the previous study by Reder et al. (5), this study investigated physical effort instead of the perceived difficulty of the tasks (NASA-TLX: from 1 to 20 points). Physical effort referred to the exertion of energy involving the entire body (e.g., leaning forward to properly view the monitor), while perceived difficulty of the tasks specifically related to the strategic challenges associated with task completion (“how the catheter had to be used”). Those were elicited using a questionnaire akin to methodologies employed in other studies (5, 17–20). Additionally, parameters were determined for the manual focus (or “manual component”; Table 1) of activities. This was assessed for both professional and non-professional activities (such as playing musical instruments, engaging in sports, and other hobbies). Activities primarily involving foot focus (e.g., jogging) were rated with 0, while activities with a clear hand focus (e.g., sewing) were rated with 11 (Table 1). Activities requiring both hand and foot coordination were rated with 5.

**Table 1:** Correlation analyses of parameters derived directly from catheter experiments and questionnaire. This table presents correlation coefficients between objective and subjective parameters from the experiment and questionnaire and the computed lifelong manual focus indices, stratified into profession‑based and hobby‑based manual foci. Hobby‑based manual foci represent the sum of all non‑professional manual activities, whereas “sum manual foci” denotes the total of all professional and non‑professional manual foci. Correlation analyses are further stratified by sex, with separate coefficients shown for female and male participants.

		Female (*n* = 25)							Male (*n* = 36)						
		No. movements	No. attempts	Pathway [in cm]	Time [in s]	Physical efforts	Self-assessed performance	Stress level	No. movements	No. attempts	Pathway [in cm]	Time [in s]	Physical efforts	Self-assessed performance	Stress level
Profession-based Manual Focus	R	0.095	0.339	0.234	0.199	0.294	−0.075	0.135	0.044	−0.175	0.060	−0.072	−0.212	0.206	−0.187
	P	0.659	0.106	0.270	0.351	0.154	0.723	0.519	0.794	0.299	0.722	0.672	0.208	0.221	0.269
Music Instrument Practice [Manual Focus]	R	−0.081	0.021	−0.016	−0.021	0.020	−0.097	0.074	−0.106	−0.226	−0.152	−0.153	−0.001	0.091	**−0.378**
	P	0.702	0.920	0.939	0.920	0.922	0.636	0.720	0.527	0.172	0.363	0.360	0.993	0.585	**0.019**
Sports Practice [Manual Focus]	R	−0.076	−0.133	−0.154	−0.209	−0.363	−0.087	0.021	−0.127	0.036	0.007	−0.022	0.071	−0.086	−0.009
	P	0.717	0.526	0.462	0.316	0.068	0.671	0.920	0.447	0.831	0.967	0.894	0.671	0.606	0.956
Other Hobbies Practice [Manual Focus]	R	0.065	0.105	0.111	0.039	−0.269	0.176	−0.248	−0.190	−0.240	−0.251	−0.192	−0.217	0.133	−0.148
	P	0.757	0.619	0.596	0.852	0.184	0.390	0.221	0.252	0.146	0.129	0.248	0.190	0.424	0.375
Hobby-based Manual Foci [Sum]	R	−0.008	0.046	0.049	−0.030	−0.243	0.037	−0.058	**−0.402**	**−0.409**	**−0.397**	**−0.342**	−0.144	0.153	**−0.443**
	P	0.971	0.827	0.816	0.888	0.232	0.856	0.780	**0.012**	**0.011**	**0.014**	**0.035**	0.387	0.360	**0.005**
Sum of Manual Foci [total]	R	0.017	0.075	0.090	−0.006	−0.148	0.024	−0.051	−0.302	**−0.440**	−0.293	−0.299	−0.082	0.159	**−0.430**
	P	0.936	0.727	0.675	0.977	0.480	0.909	0.807	0.069	**0.006**	0.078	0.072	0.630	0.347	**0.008**

Significant correlations (*p* < 0.05 with 95% confidence intervals) and their corresponding correlation coefficients R are highlighted in bold.

The over-lifetime potentiated professional manual focused performance (“Total Professional Performance Level” or “tPPL”, see Equation 1) was calculated using the Profession-based Manual Focus (MF_Prof_), the hours per day using Manual Focus (MF_HD_), the Profession Practice in years (PP_y_), and in hours per week (PP_HW_):


$$\text{tPPL}=MF_{\text{Prof}}*PP_{Y}*MF_{\mathrm{HD}}*52\;\text{Weeks}*PP_{\mathrm{HW}}$$ (1)

The over-lifetime potentiated manual focused performance regarding non-professional activities (“Total Non-Professional Performance Level” or “tNPPL_Hobby_”) was calculated using the Activity-based Manual Focus (e.g., MF_Music_ or MF_Sport_), the practice duration in years (HP_y_), and hours per week for practicing this hobby (HP_HW_), as depicted for “tNPPL_Sport_” in Equation 2:


$$\text{tNPP}L_{\text{Sport}}=MF_{\text{Sport}}*HP_{Y\;(\text{Sport})}*52\;\text{Weeks}*HP_{\mathrm{HW}\;(\text{Sport})}$$ (2)

The three hobby-based performance levels were aggregatable due to their standardized dimensionality (“Hobby-based Manual Foci [Sum]”). For further value comparability, both subjective sum parameters underwent z-transformation. Subsequently, these adjusted values could be combined into a sum score (“Total Subjective Performance” or “TSP”), facilitating comparability with the sum score of the objective parameters (TOP).

### Statistical analysis

Using the statistical software SPSS (Version 29, IBM), Dunnett’s Multi-Comparison Test was employed to compare the mean values of sex-specific objective parameters (2-sided; *α* = 0.05). Correlations between measured parameters and subjective stress levels, assessed difficulty, and performance were analyzed using a Spearman Test (partial ordinal scaled parameters). In case of nominal scaled parameters, the Fisher Test was applied (or “exact Chi Square Test” with Monte Carlo Significance Test and 99% CI). Multiple linear regression analyses with backward elimination and stepwise variable selection were used to predict subjective parameters and the self-assessed performance (F probability of inclusion is set at *p* ≤ 0.05, and the probability of exclusion is set at *p* ≥ 0.07). In this process, the regression coefficients (R, B, and standardized *β*; including *p*-values, the determination coefficient R^2^ or “R squared”) were provided. Only regression models with predictors with statistically significant influences on the regression equations were reported (*p* < 0.05). Multicollinearity was considered (if r > 0.9; *p* < 0.05) (21, 22).

## Results

Data were collected from 64 participants (26 female; right-handed > 95% each with *p* = 0.65), with one female participant withdrawing due to perceived stress levels (*n* = 63) (5). No significant differences were observed among the three main profession categories (medical, crafts, administrative; *p* = 0.584), or sex-specific age categories (*p* = 0.129; Supplementary Table 1) (5). There was no sex-specific differences in the hours of manual work per day during professional activities (*p* = 0.526), in the weekly hours dedicated to playing music (*p* = 0.463), participating in sports (*p* = 0.119), or pursuing other hobbies (*p* = 0.611; Supplementary Table 1). However, there were significant differences in the self-reported weekly working hours, with *n* = 24 men and *n* = 6 women reporting a working time >40 h (*p* = 0.025).

### Objective study parameters

From the previous study (5) it is already known, that men required less time to solve the tasks throughout the entire experiment setup (502 ± 230.19 s) compared to women (688.8 ± 364 s; *p* = 0.019; Supplementary Table 2). Additionally, men took less time to comprehend the instructions than women (25.4 ± 112.4 s vs. 102.5 ± 208.6 s; *p* = 0.02) and asked for help less frequently (0, IQR 0/0 vs. 0, IQR 0/5; *p* = 0.02; Supplementary Table 2) (5). Regarding the median TOP, there were no significant differences between women and men (54,080, IQR 10,920 / 99,840 vs. 66,560, IQR 13,000 / 153,920; *p* = 0.707).

### Subjective study parameters

There were no significant differences between women and men in the reported manual focus for the hobby-based activities (*p* = 0.269 to 0.792), as was also the case for the profession-related manual focus, observing a tendency in favor of men with 6 (IQR 3/6) vs. 6 (IQR 2.5/6; *p* = 0.062; Supplementary Table 1). The median TSP in women and men did not differ significantly (7,020, IQR 2,730/9,633 vs. 6,552, IQR 4,212/9,542; *p* = 0.08).

### Correlation between objective and subjective parameters

In men, the manual focus associated with playing music instruments was negatively correlated with stress levels (*r* = −0.378; *p* = 0.019; Table 1). Specifically, the sum score of non-professional activities (“Hobby-based Manual Foci [Sum]” or tPPL) correlated strongly negatively with parameters such as NoM, NoA, Pathway, Time, and Self-assessed Stress Level (*r* = −0.342 to −0.443; *p* = 0.035 to 0.005; Table 1). Profession-related manual focus had no significant effect on all objective and subjective parameters. The total score of all manual foci only correlated negatively with NoA and stress levels (*r* = −0.440 and −0.430; *p* = 0.006 and 0.008; Table 1). In women, there was no significant correlation observed overall between objective and subjective parameters.

Hobby-related manual activities (or tNPPL) had a comparable effect on TSP in both women and men (r > 0.72 and *p* < 0.001 each; Table 2). However, tPPL had a lower impact on women compared to men (*r* = 0.406 vs. r = 0.773; *p* = 0.044 vs. *p* < 0.001). The influence of TSP on TOP ultimately increased only in men, unlike women (*r* = 0.364; *p* = 0.027 vs. r = −0.025; *p* = 0.91). It is noteworthy that this increase seemed to be driven by a large effect of tNPPL (*r* = 0.566; *p* < 0.001), whereas tPPL had no significant influence on TSP (Table 2). In women, only the self-assessed performance had an influence on the TOP (*r* = 0.488; *p* = 0.013), while neither tNPPL nor tPPL did. In men, the reported stress level significantly correlated negatively with all objective and subjective parameters (Table 2). Due to these significant relationships of self-assessed performance and stress level with sex-specific differences, these factors were considered in the subsequent regression analysis.

**Table 2:** Correlation of lifelong manual-focused performances from professional and leisure activities with the calculated objective experiment performance, as well as physical efforts and self-assessment parameters from the NASA task load index.

Sex		Female	Male	Female	Male	Female	Male	Female	Male	Female	Male
Variables		Total subjective performance	Total subjective performance	Total objective performance	Total objective performance	Self-assessed physical efforts	Self-assessed physical efforts	Self-assessed performance	Self-assessed performance	Self-assessed stress level	Self-assessed stress level
Total professional performance level	R	**0.406**	**0.773**	0.026	0.062	0.238	−0.188	0.045	−0.011	0.102	**−0.349**
	P	**0.044**	**<0.001**	0.905	0.715	0.253	0.264	0.831	0.949	0.628	**0.034**
Total non-professional performance level	R	**0.759**	**0.725**	−0.021	**0.566**	−0.328	−0.176	0.067	−0.049	−0.161	**−0.493**
	P	**<0.001**	**<0.001**	0.921	**<0.001**	0.102	0.291	0.745	0.771	0.433	**0.002**
Total subjective performance	R	1.000	1.000	−0.025	**0.364**	−0.118	−0.258	0.032	−0.046	−0.159	**−0.557**
	P			0.907	**0.027**	0.573	0.124	0.881	0.786	0.449	**<0.001**
Total objective performance	R	−0.025	**0.364**	1.000	1.000	0.023	−0.180	**0.488**	0.077	−0.182	**−0.491**
	P	0.907	**0.027**			0.912	0.281	**0.013**	0.644	0.384	**0.002**

Significant correlations (*p* < 0.05 with 95% confidence intervals) and their corresponding correlation coefficients R are highlighted in bold.

### Multiple linear regression analyses

In men and women, there are significant differences in predicting TOP based on the TSP. Particularly, tNPPL was strongly positively correlated with achieved objective performance in men (Model 1 in Table 3; *β* = 0.526; *p* = 0.001), unlike in women. In fact, the tPPL strongly negatively correlated with the achieved TOP in men (Model 2 in Table 3; *β* = −0.579; *p* = 0.022). In women, subjective hand-focused activities (TSP, tNPPL, tPPL; Model 1 and 2 in Table 3) overall did not significantly correlate with the achieved TOP. However, in women, self-assessed performance strongly correlated with the objective performances (Model 3 and 4 in Table 4; *β* > 0.5 and *p* < 0.01 each). An associated influence of stress levels could not be demonstrated in women (Model 5–7 in Supplementary Table 3; *p* > 0.05), unlike in men. Here, stress levels strongly negatively correlated with the objectively achieved performance TOP (Model 5–7 in Supplementary Table 3; *β* = −0.46 to −0.56; *p* < 0.01 each). For a statistical power of 1-β = 0.9 in a cohort of *n* = 63 participants, with two predictors, and an alpha level of *α* = 0.05, regression analyses should achieve a minimum R^2^ = 0.174 (R^2^ = 0.194 with three predictors) (23, 24). The sample size and number of predictors were deemed sufficient for conducting a meaningful analysis, providing adequate statistical power to derive reliable conclusions from the data.

**Table 3:** Sex-specific multiple linear regression analyses on manual-focused performance from non-professional and professional activities.

To predict: total objective performance (TOP)				Coeff. B	Std. β	*p*	B (Lower 95% CI)	B (Upper 95% CI)	R	R^2^
Model 1	Male	1	Const.	0.732		0.099	−0.145	1.608	0.528	0.278
			Total Professional Performance Level	0.098	0.037	0.803	−0.696	0.892		
			Total Non-Professional Performance Level	1.636	0.521	0.001	0.699	2.574		
		2	Const.	0.748		0.085	−0.107	1.602	0.526	0.277
			Total Non-Professional Performance Level	1.652	0.526	0.001	0.736	2.567		
	Female	1	Const.	−0.971		0.329	−2.990	1.048	0.252	0.064
			Total Professional Performance Level	0.979	0.168	0.465	−1.755	3.713		
			Total Non-Professional Performance Level	−0.559	−0.139	0.543	−2.441	1.322		
		2	Const.	−0.907		0.351	−2.881	1.067	0.216	0.047
			Total Professional Performance Level	1.261	0.216	0.311	−1.260	3.782		
		3	Const.	−1.202		0.199	−3.084	0.680	0.0	0.0
Model 2	Male	1	Const.	0.732		0.099	−0.145	1.608	0.528	0.278
			Total Professional Performance Level	−1.538	−0.579	0.022	−2.844	−0.233		
			Total Subjective Performance	1.636	0.858	0.001	0.699	2.574		
	Female	1	Const.	−0.971		0.329	−2.990	1.048	0.252	0.064
			Total Professional Performance Level	1.538	0.263	0.254	−1.190	4.267		
			Total Subjective Performance	−0.559	−0.139	0.543	−2.441	1.322		
		2	Const.	−0.907		0.351	−2.881	1.067	0.216	0.047
			Total Professional Performance Level	1.261	0.216	0.311	−1.260	3.782		
		3	Const.	−1.202		0.199	−3.084	0.680	0.0	0.0

**Table 4:** Sex-specific multiple linear regression analyses on manual-focused performance from non-professional and professional activities, along with the self-assessed performance.

To predict: total objective performance (TOP)				Coeff. B	Std. β	*p*	B (Lower 95% CI)	B (Upper 95% CI)	R	R^2^
Model 3	Male	1	Const.	−0.548		0.739	−3.861	2.764	0.414	0.171
			Total Subjective Performance	0.770	0.403	0.014	0.163	1.376		
			Self-assessed Performance	0.107	0.122	0.440	−0.172	0.387		
		2	Const.	0.672		0.152	−0.260	1.604	0.396	0.157
			Total Subjective Performance	0.755	0.396	0.015	0.154	1.356		
	Female	1	Const.	−8.313		0.003	−13.410	−3.216	0.556	0.309
			Total Subjective Performance	−0.638	−0.158	0.401	−2.186	0.910		
			Self-assessed Performance	0.767	0.565	0.006	0.245	1.289		
		2	Const.	−7.786		0.003	−12.677	−2.896	0.534	0.285
			Self-assessed Performance	0.725	0.534	0.007	0.217	1.232		
Model 4	Male	1	Const.	−0.718		0.631	−3.728	2.291	0.558	0.311
			Total Non-Professional Performance Level	1.731	0.551	0.000	0.834	2.628		
			Self-assessed Performance	0.126	0.141	0.325	−0.130	0.381		
		2	Const.	0.704		0.095	−0.130	1.538	0.54	0.292
			Total Non-Professional Performance Level	1.697	0.540	0.000	0.804	2.591		
	Female	1	Const.	−7.669		0.002	−12.292	−3.046	0.576	0.331
			Total Non-Professional Performance Level	−1.054	−0.262	0.150	−2.520	0.411		
			Self-assessed Performance	0.727	0.544	0.005	0.241	1.213		
		2	Const.	−7.299		0.004	−12.002	−2.596	0.514	0.264
			Self-assessed Performance	0.686	0.514	0.009	0.191	1.180		

## Discussion

This study aimed to examine the influence of leisure activities, fine motor skills, and psychological factors on objective performance during early interventional radiology (IR) training, with a particular focus on sex-specific differences. A total of 63 participants (26 females) were included to explore how manual focus in both professional and non-professional activities impacted performance in a catheterization task. In men, non-professional activities were negatively correlated with self-reported stress levels, suggesting that diverse leisure activities might offer benefits. No significant sex-specific differences were observed in manual focus or perceived stress levels. For women, objective performance appeared to be primarily influenced by self-assessed performance, whereas in men, stress levels negatively impacted performance. Additionally, non-professional activities were positively correlated with performance in men, while professional activities showed no significant effect. Multiple regression analyses further supported the hypothesis that non-professional activities enhance male performance, while women’s performance seemed more influenced by self-assessment. Overall, the findings largely confirmed the proposed hypotheses regarding sex-specific differences in performance drivers.

In male participants’, fine motor skills - particularly those developed through leisure activities - positively influenced their objective performance. This highlights the potential benefits of a balanced lifestyle for men, where non-professional activities like playing musical instruments may foster manual dexterity and coordination in ways that professional training might not. These findings aligned with prior research, emphasizing the role of experiential learning in developing personal competencies (25–27), potentially offering a more significant impact than professional training alone (28–33). Conversely, female participants’ fine motor skills, irrespective of their source, did not correlate significantly with objective performance. Instead, their self-assessed performance played a critical role in determining their outcomes (34, 35). This suggested that psychological factors, particularly self-esteem and confidence, are more influential in women’s performance than in men’s (34–36). Women may benefit from psychosocial support to enhance their performance and avoid unjustified criticism (16, 34–36). It can be hypothesized that men’s performance could be influenced by their confidence in their abilities (37, 38), while women’s performance seemed more deeply tied to their internal processes of self-assessment (2, 5).

**Figure 1:**
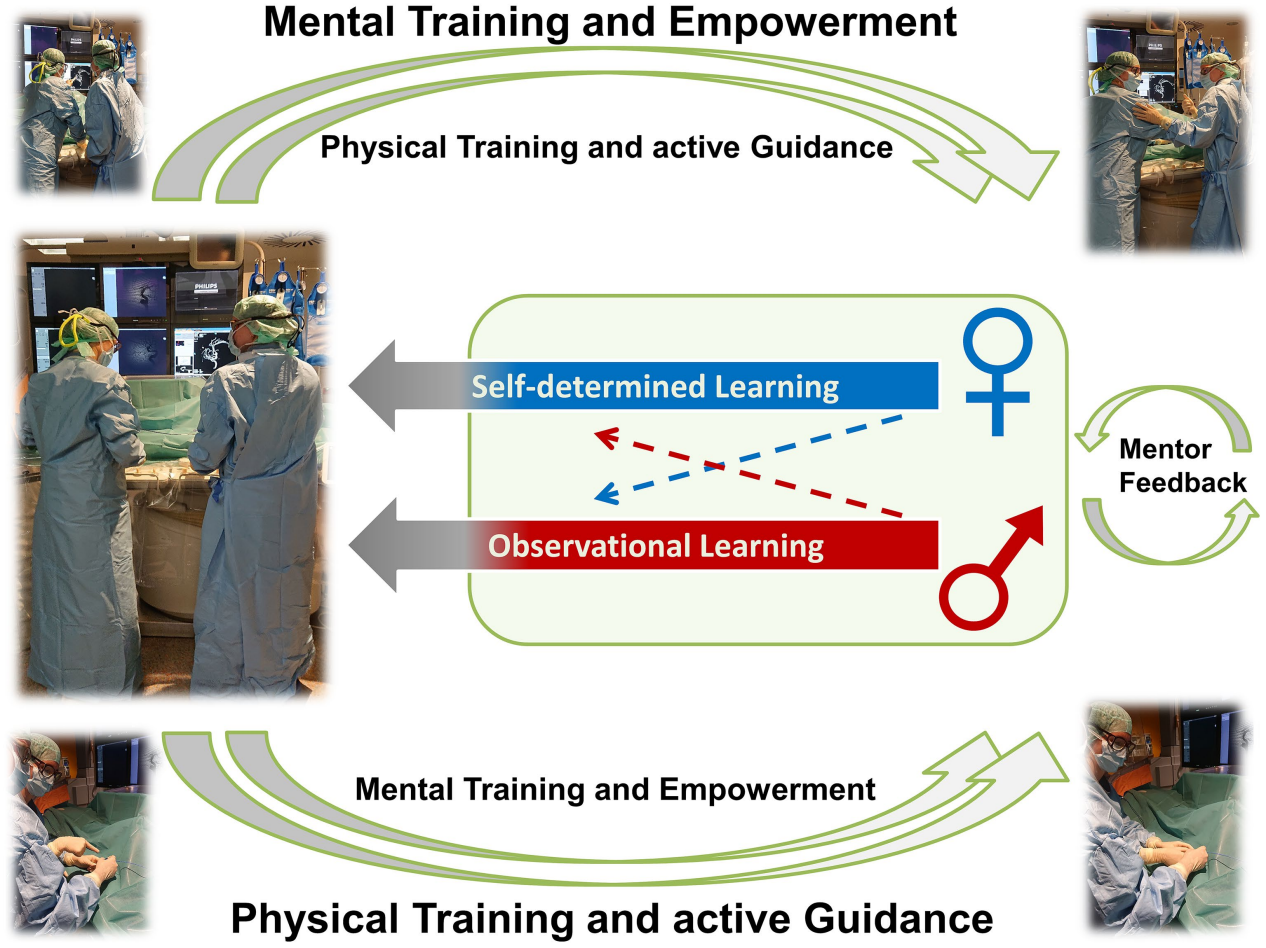
Sex-specific learning patterns suggest that women tend to benefit more from mental training, constructive verbal consolidation of learned material, and empowerment, whereas men tend to benefit more from physical training and active guidance.

These observations underscore the significant role of psychological factors in influencing women’s performance, a conclusion supported by Reder et al. (5), presuming that women were more accurate in assessing their objective performance than men (Figure 1). Given these findings, women may benefit from psychosocial support aimed at boosting self-esteem and counteracting self-doubt, possibly improving both their performance and satisfaction with training.

For men, an externally guided learning approach—particularly one that builds on their intrinsic motivation—may yield greater success, as suggested by previous research (2). This external guidance could complement their inclination to perform tasks independently and avoid seeking help, which was observed in this study and reported previously (5). In contrast, women seemed to benefit from a learning style that combines external motivation with intrinsic direction and task execution, evidenced by their tendency to seek help more often and spend more time comprehending instructions (5, 7–10). Interestingly, while women generally made fewer mistakes than men (5), this may be due to their more careful approach and desire to understand the material thoroughly (39).

In both sexes, feedback appears to be a key component for performance improvement, supporting the idea that constructive feedback is essential for developing both mental and physical skills (40–42). However, the gendered differences in response to feedback, self-assessment, and stress levels suggested that future research should consider how these factors interact with learning outcomes and task performance. Additionally, future studies should explore how other demographic factors, such as gender identity and socioeconomic status, may influence task performance and learning strategies. Overall, these findings highlight the importance of considering sex-specific differences when designing educational and occupational strategies. Tailoring approaches to the individual needs and learning preferences of both men and women could enhance fine motor performance, reduce stress, and improve satisfaction in educational settings and the workforce. Further studies are needed to explore the impact of other gender identities, including non-binary and intersex individuals, on learning behaviors, which could offer a more comprehensive understanding of these issues.

Several limitations must be acknowledged. One of the primary limitations was the relatively small sample size of 63 participants (26 female), constraining the generalizability of the results. A larger sample size would have increased statistical power and allowed for more reliable conclusions. Additionally, although standardized teaching methods were employed, a male medical student was involved in the recruitment, training, and execution of the experiments. This could have introduced gender-related bias, as the personality and gender of the experimenter might have influenced the behavior of the participants. Male participants might have felt more confident in demonstrating their skills or seeking help, while female participants might have been less likely to openly acknowledge difficulties due to social or cultural factors. Another limitation arose from the selection of participants with limited experience in catheterization. The study primarily targeted the early stages of learning, while the results might have been different for well-trained, experienced interventional specialists. However, defining “well-trained” individuals presented a challenge, as training levels varied greatly among individuals and institutions. By choosing inexperienced participants, potential confounding factors related to varying training levels were minimized, allowing the study to focus on the learning process during the early stages of training. It was also important to note that this study only investigated learning within a 90-min timeframe. This short duration of training likely did not capture the long-term effects of continuous training or the gradual development of fine motor skills that might have occurred with prolonged engagement in the task. Therefore, the results likely reflected short-term learning outcomes rather than the long-term effects of sustained practice. Future studies could extend the training duration to explore the impact of prolonged learning over weeks or months, providing deeper insights into the long-term learning process. Despite these limitations, the use of data from the previous study from Reder et al. (5) significantly strengthened the validity of the current investigation. Since the previously published findings formed the basis of the current results, and the new data merely augmented the previous findings, there was no concern regarding the introduction of bias or variability from a new cohort. This approach eliminated the need to account for the potential confounding effects of a new participant group, thereby enhancing the validity and relevance of the current study. Rather than introducing a new cohort, the findings of the prior study were extended by the current data, reinforcing the consistency and robustness of the results.

In conclusion, this study highlighted the importance of considering sex-specific differences in learning strategies and fine motor skill development. Men appeared to benefit from a varied, experience-based approach to learning that incorporated leisure activities, which could have enhanced their motor skills. In contrast, women’s performance seemed to be more influenced by self-assessment and psychological factors such as self-esteem. Tailoring training methods to account for these differences could have improved performance and satisfaction for both men and women in educational and professional settings. Further studies should have examined the influence of additional variables, such as personality, motivation, and gender identity, on learning behavior and performance outcomes. These findings may have contributed to the development of more effective and inclusive educational strategies that lead to better learning outcomes across diverse groups.

### Additional informations

#### Transparency statement

All authors confirm that the present manuscript represents a transparent and honest account of the reported research. This investigation is directly related to a previous study conducted by the same primary authors, entitled “Gender differences in self-assessed performance and stress level during training of basic interventional radiology maneuvers” (DOI: https://doi.org/10.1007/s00330-023-09993-3). In that prior study, 64 participants, all equally inexperienced in neurointerventional techniques, underwent standardized training and were required to complete predefined tasks within a silicon vascular model. The movements of both catheters and microguidewires inside the model were analyzed and compared with subjective ratings of perceived workload, stress, and performance (using the NASA Task Load Index, NASA-TLX). The so-called ‘hand focus’ during both leisure activities and professional tasks was captured solely as a single, independent component to identify any potential confounder. In mediation analyses from the previous study, ‘hand focus’ did not significantly affect observed objective performance measures.

Given the highly subjective nature of these self-reported ‘hand focus’ values (“How high is your hand focus at work?”; “How high is your hand focus during leisure activities?”), an additional methodology was conceptualized to further objectify this parameter. The underlying hypothesis posited that fine motor skills can be trained, and that long-term engagement in such activities should be reflected in greater self-confidence, potentially impacting perceived performance, stress, etc. (in a positive direction). A formula was developed to represent the ‘lifelong acquired hand focus’ (here, fine motor skills) as a composite, cumulatively weighted parameter—stratified according to skills gained either professionally or through leisure activities. These parameters were then analyzed via correlation and regression with the objective primary metrics of the initial study (e.g., time to task completion, catheter path length) as well as subjective NASA-TLX scores.

Results from the previous study are strictly marked as already published data in the present manuscript. For clarity, the results of the initial study are summarized in the Supplementary materials as separate figures and tables, enabling comprehensive insight into the relevant body of work discussed. To ensure direct comparability and expand upon the statements of the first study, the original dataset was re-analyzed to address the new research questions. Thus, there is substantial overlap in the description of the experimental procedures and the evaluation of parameters derived from both the practical tasks and the questionnaire responses. Furthermore, both studies examine the same cohort, avoiding bias due to different subject pools, and providing a unified response to the overarching question: are there further gender-based differences in motor learning?

Key new findings include evidence that excessive weekly professional workload may impair fine motor skill development, particularly among men, while a lifelong process of acquiring such skills appears less beneficial for motor learning among women than men (a novel finding of the current investigation). As previously shown, women better estimated their own objective performance (as consistently reported in the first study). Combining both studies, the overarching finding emerges that women may benefit more from mental support and resilience-building interventions, while men profit from motor training but seem to rely on honest external feedback for accurate self-assessment.

## Data Availability

The raw data supporting the conclusions of this article will be made available by the authors, without undue reservation.
