# Supplementary material for: Impact of fine motor skills acquisition and psychological factors on sex-specific performance in early interventional radiology training
Source: Front Med (Lausanne). 2025 Dec 5;12:1638221. doi: 10.3389/fmed.2025.1638221 (PMC12714640; doi:10.3389/fmed.2025.1638221)
Supplement: Supplementary file 1 [file Table_1.DOCX]

**Supplementary Table 3:** Sex-specific Multiple Linear Regression Analyses on Manual-focused Performance from Non-professional and Professional Activities, as well as the Self-assessed Stress Level.

| To predict: Total Objective Performance (TOP) | | | | Coeff. B | Std. β | p | B  (Lower 95% CI) | B  (Upper 95% CI) | R | R² |
| --- | --- | --- | --- | --- | --- | --- | --- | --- | --- | --- |
| Model 5 | **male** | 1 | Const. | 3.126 |  | 0.001 | 1.436 | 4.815 | 0.607 | 0.368 |
|  |  |  | Total Subjective Performance | 0.155 | 0.081 | 0.626 | -0.485 | 0.795 |  |  |
|  |  |  | Self-assessed Stress Level | -0.376 | -0.557 | 0.002 | -0.602 | -0.150 |  |  |
|  |  | 2 | Const. | 3.352 |  | 0.000 | 1.961 | 4.743 | 0.603 | 0.364 |
|  |  |  | Self-assessed Stress Level | -0.407 | -0.603 | 0.000 | -0.592 | -0.222 |  |  |
|  |  |  | Total Subjective Performance | 0.155 | 0.081 | 0.626 | -0.485 | 0.795 |  |  |
|  |  |  | Self-assessed Stress Level | -0.376 | -0.557 | 0.002 | -0.602 | -0.150 |  |  |
|  | **female** | 1 | Const. | -0.771 |  | 0.708 | -4.998 | 3.456 | 0.076 | 0.006 |
|  |  |  | Total Subjective Performance | -0.260 | -0.065 | 0.777 | -2.149 | 1.629 |  |  |
|  |  |  | Self-assessed Stress Level | -0.056 | -0.060 | 0.792 | -0.491 | 0.380 |  |  |
|  |  | 2 | Const. | -1.247 |  | 0.203 | -3.217 | 0.722 | 0.049 | 0.002 |
|  |  |  | Total Subjective Performance | -0.196 | -0.049 | 0.821 | -1.975 | 1.582 |  |  |
|  |  | 3 | Const. | -1.202 |  | 0.199 | -3.084 | 0.680 | 0.0 | 0.0 |
| Model 6 | **male** | 1 | Const. | 2.679 |  | 0.001 | 1.208 | 4.149 | 0.67 | 0.499 |
|  |  |  | Total Non-Professional Performance Level | 0.967 | 0.308 | 0.041 | 0.040 | 1.894 |  |  |
|  |  |  | Self-assessed Stress Level | -0.310 | -0.460 | 0.003 | -0.509 | -0.111 |  |  |
|  | **female** | 1 | Const. | -0.332 |  | 0.869 | -4.457 | 3.792 | 0.22 | 0.048 |
|  |  |  | Total Non-Professional Performance Level | -0.931 | -0.231 | 0.305 | -2.770 | 0.908 |  |  |
|  |  |  | Self-assessed Stress Level | -0.091 | -0.102 | 0.648 | -0.497 | 0.316 |  |  |
|  |  | 2 | Const. | -1.155 |  | 0.200 | -2.965 | 0.655 | 0.198 | 0.039 |
|  |  |  | Total Non-Professional Performance Level | -0.795 | -0.198 | 0.344 | -2.497 | 0.906 |  |  |
|  |  | 3 | Const. | -1.156 |  | 0.198 | -2.959 | 0.648 | 0.0 | 0.0 |
| Model 7 | **male** | 1 | Const. | 2.679 |  | 0.001 | 1.208 | 4.149 | 0.67 | 0.499 |
|  |  |  | Total Non-Professional Performance Level | 0.967 | 0.308 | 0.041 | 0.040 | 1.894 |  |  |
|  |  |  | Self-assessed Stress Level | -0.310 | -0.460 | 0.003 | -0.509 | -0.111 |  |  |
|  | **female** | 1 | Const. | -0.332 |  | 0.869 | -4.457 | 3.792 | 0.22 | 0.048 |
|  |  |  | Total Non-Professional Performance Level | -0.931 | -0.231 | 0.305 | -2.770 | 0.908 |  |  |
|  |  |  | Self-assessed Stress Level | -0.091 | -0.102 | 0.648 | -0.497 | 0.316 |  |  |
|  |  | 2 | Const. | -1.155 |  | 0.200 | -2.965 | 0.655 | 0.198 | 0.039 |
|  |  |  | Total Non-Professional Performance Level | -0.795 | -0.198 | 0.344 | -2.497 | 0.906 |  |  |
|  |  | 3 | Const. | -1.156 |  | 0.198 | -2.959 | 0.648 | 0.0 | 0.0 |
