# Supplementary material for: Impact of fine motor skills acquisition and psychological factors on sex-specific performance in early interventional radiology training
Source: Front Med (Lausanne). 2025 Dec 5;12:1638221. doi: 10.3389/fmed.2025.1638221 (PMC12714640; doi:10.3389/fmed.2025.1638221)
Supplement: Supplementary file 2 [file Table_2.DOCX]

**Supplementary Table 1: Basic Population Characteristics.**

|  |  | Female | Male |  |
| --- | --- | --- | --- | --- |
|  | **Subgroups** | **Median (IQR)/**  **N (%)** | **Median (IQR)/**  **N (%)** | **p** |
| N (Sex) | Female/Male | 26 (41%) | 38 (59%) | - |
| Age Categories^†^ | 20-30 | 13 (50%) | 12 (31.6%) | 0.129 |
|  | 31-40 | 5 (19.2%) | 18 (47.4%) |  |
|  | 41-50 | 4 (15.4%) | 5 (13.2%) |  |
|  | 51-60 | 4 (15.4%) | 3 (7.9%) |  |
| Handedness^†^ | Right | 25 (96.2%) | 37 (97.4%) | 0.65 |
|  | Left | 1 (3.8%) | 0 (0%) |  |
|  | Both-sided | 0 (0%) | 1 (2.6%) |  |
| Profession^†^ | Medical | 13 (50%) | 24 (63.2%) | 0.584 |
|  | Crafts | 1 (3.8%) | 1 (2.6%) |  |
|  | Administrative | 12 | 13 (34.2%) |  |
| Profession Practice [Years] |  | 12.5 (10/20) | 15 (10/15) | 0.127 |
| Profession Practice [Hours/Week] | <10 | 1 (3.8%) | 1 (2.6%) | 0.025 |
|  | 10-20 | 3 (11.5%) | 2 (5.3%) |  |
|  | 21-30 | 4 (15.4%) | 1 (2.6%) |  |
|  | 31-40 | 12 (46.2%) | 10 (26.3%) |  |
|  | 41-50 | 1 (3.8%) | 8 (21.1%) |  |
|  | >50 | 5 (19.2%) | 16 (42.1%) |  |
| Profession-based Manual Component [Hours/Day]^†^ |  | 1 (0/3.25) | 1.50 (0/4) | 0.526 |
| Profession-based Manual Focus | 0-11 | 6 (4.5/8) | 4 (2/6) | 0.062 |
| Music Instrument Practice [Years] |  | 0 (0/6) | 4 (4/6) | 0.396 |
| Music Instrument Practice [Hours/Week] |  | 0 (0/1) | 1 (0/1) | 0.463 |
| Music Instrument Practice [Manual Focus]^†^ | 0-11 | 8 (0/10) | 0 (0/9) | 0.792 |
| Sports Practice [Years] |  | 6 (3/6) | 6 (2.5/6) | 0.063 |
| Sports Practice [Hours/Week] |  | 2 (2/2.25) | 2 (1/2.25) | 0.119 |
| Sports Practice [Manual Focus]^†^ | 0-11 | 6 (3/6) | 6 (5/6) | 0.269 |
| Other Hobbies Practice [Years] |  | 3.5 (0/6) | 2 (0/6) | 0.681 |
| Other Hobbies Practice [Hours/Week] |  | 0.50 (0/2) | 0.5 (0/2) | 0.611 |
| Other Hobbies Practice [Manual Focus]^†^ | 0-11 | 3 (0/10) | 3 (0/10) | 0.721 |
| Sum of Hobby-based Manual Foci | 0-33 | 14 (6/21.25) | 14 (10/17.25) | 0.349 |
| Sum of all Manual Foci | 0-44 | 17 (10/27) | 20 (14.5/24) | 0.262 |
| *^†^From: Reder et al., Gender differences in self-assessed performance and stress level during training of basic interventional radiology maneuvers. Eur Radiol 34, 308–317 (2024), Table 1. https://doi.org/10.1007/s00330-023-09993-3* | | | | |
