# Supplementary material for: Impact of fine motor skills acquisition and psychological factors on sex-specific performance in early interventional radiology training
Source: Front Med (Lausanne). 2025 Dec 5;12:1638221. doi: 10.3389/fmed.2025.1638221 (PMC12714640; doi:10.3389/fmed.2025.1638221)
Supplement: Supplementary file 3 [file Table_3.DOCX]

**Supplementary Table 2:** Results across all tasks were consistent with those reported by Reder et al. (2024).

|  | Mean/Median/N | ±SD/IQR | Mean/Median/N | ±SD/IQR | p |
| --- | --- | --- | --- | --- | --- |
| N (Female/Male) | 25 |  | 38 |  |  |
| Time [in s]^†^ | 688.8 | ±363.95 | 501.98 | ±230.06 | 0.019 |
| Number of Movements^†^ | 1876 | 1338/2765 | 1678.5 | 1206/2209 | 0.261 |
| Number of Attempts^†^ | 71 | 34.5/90.5 | 51.5 | 39.5/69.25 | 0.121 |
| Pathways [in cm]^†^ | 275.82 | ±134.74 | 220.43 | ±100.10 | 0.097 |
| Requests for Assistance [Number]^†^ | 0 | 0/5 | 0 | 0/0 | 0.02 |
| Time to comprehend [in s]^†^ | 102.54 | ±208.56 | 25.42 | ±112.36 | 0.02 |
| Self-assessed Physical Efforts | 8.38 | ±4.78 | 6.37 | ±4.19 | 0.093 |
| Self-assessed Stress Level^†^ | 8.92 | ±4.88 | 6.29 | ±4.40 | 0.037 |
| Self-assessed Performance^†^ | 9.12 | ±3.30 | 11.32 | ±3.32 | 0.009 |
| ^†^From: Reder et al. (2024), Gender differences in self-assessed performance and stress level during training of basic interventional radiology maneuvers. Eur Radiol 34, 308–317 (2024), Table 2. https://doi.org/10.1007/s00330-023-09993-3 | | | | | |
