# Supplementary figures and images for: Impact of fine motor skills acquisition and psychological factors on sex-specific performance in early interventional radiology training
Source: Front Med (Lausanne). 2025 Dec 5;12:1638221. doi: 10.3389/fmed.2025.1638221 (PMC12714640; doi:10.3389/fmed.2025.1638221)

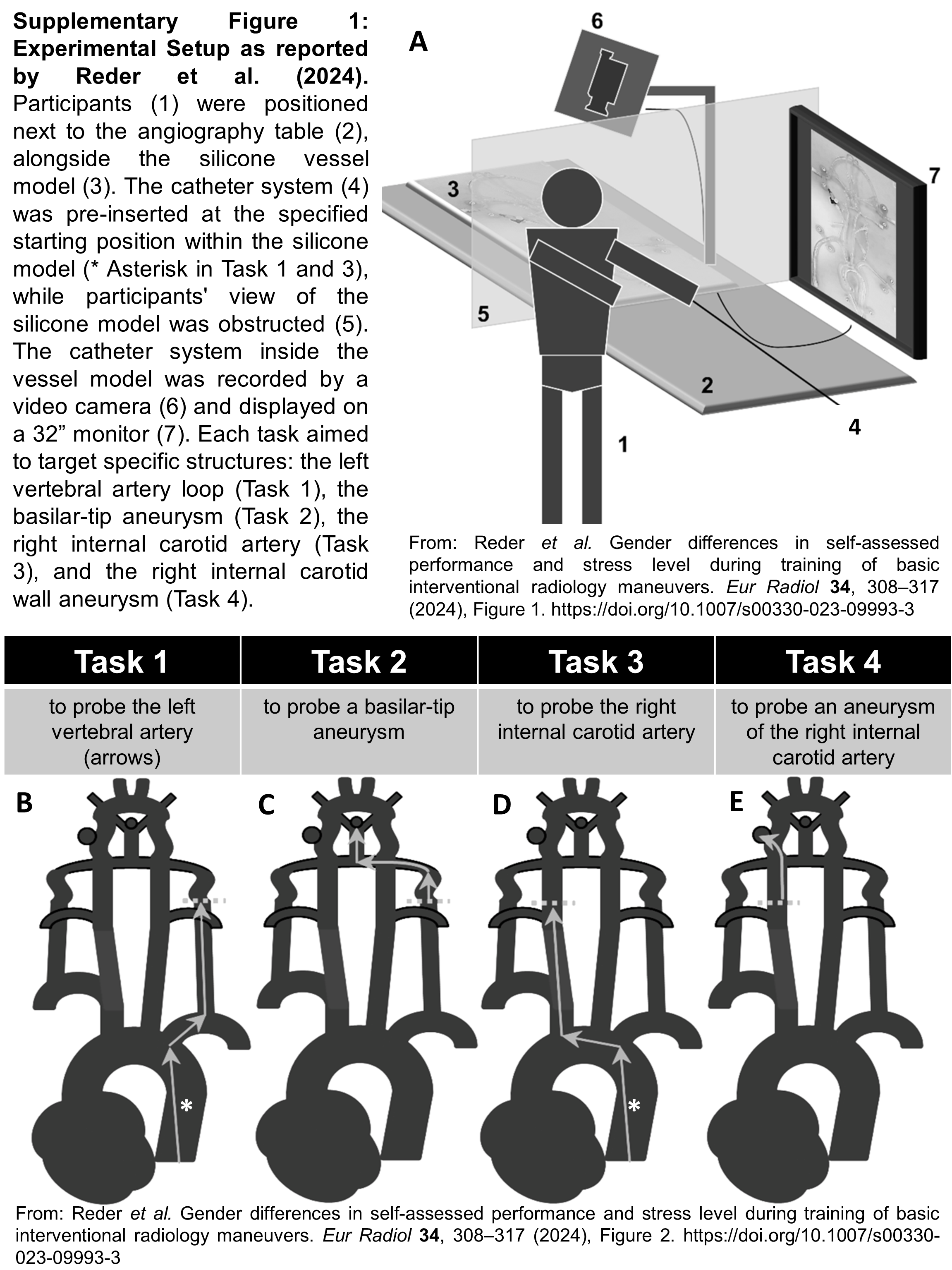

Supplement: Supplementary file 4 [file Image_1.TIF]
